# Supplementary material for: Optogenetic engineering of STING signaling allows remote immunomodulation to enhance cancer immunotherapy
Source: Nat Commun. 2023 Sep 6;14:5461. doi: 10.1038/s41467-023-41164-2 (PMC10482946; doi:10.1038/s41467-023-41164-2)
Supplement: Supplementary file 1 — Supplementary Information [file 41467_2023_41164_MOESM1_ESM.pdf]

Supplementary Information

Optogenetic engineering of STING signaling allows remote immunomodulation to enhance cancer immunotherapy

Yaling Dou[1,*], Rui Chen[1,*], Siyao Liu[1], Yi-Tsang Lee[1], Ji Jing[1], Xiaoxuan Liu[1], Yuepeng Ke[1], Rui Wang[1], Yubin Zhou[1,2,#], and Yun Huang[1,2,#]

[1]Institute of Biosciences and Technology, Texas A&M University, Houston, TX, USA

[2]Department of Translational Medical Sciences, School of Medicine, Texas A&M University, Houston, TX 77030, USA

Corresponding authors: Yubin Zhou, yubinzhou@tamu.edu; Yun Huang, yun.huang@tamu.edu

The PDF file includes:

Supplementary Figures 1 to 12

Supplementary Tables 1 to 4

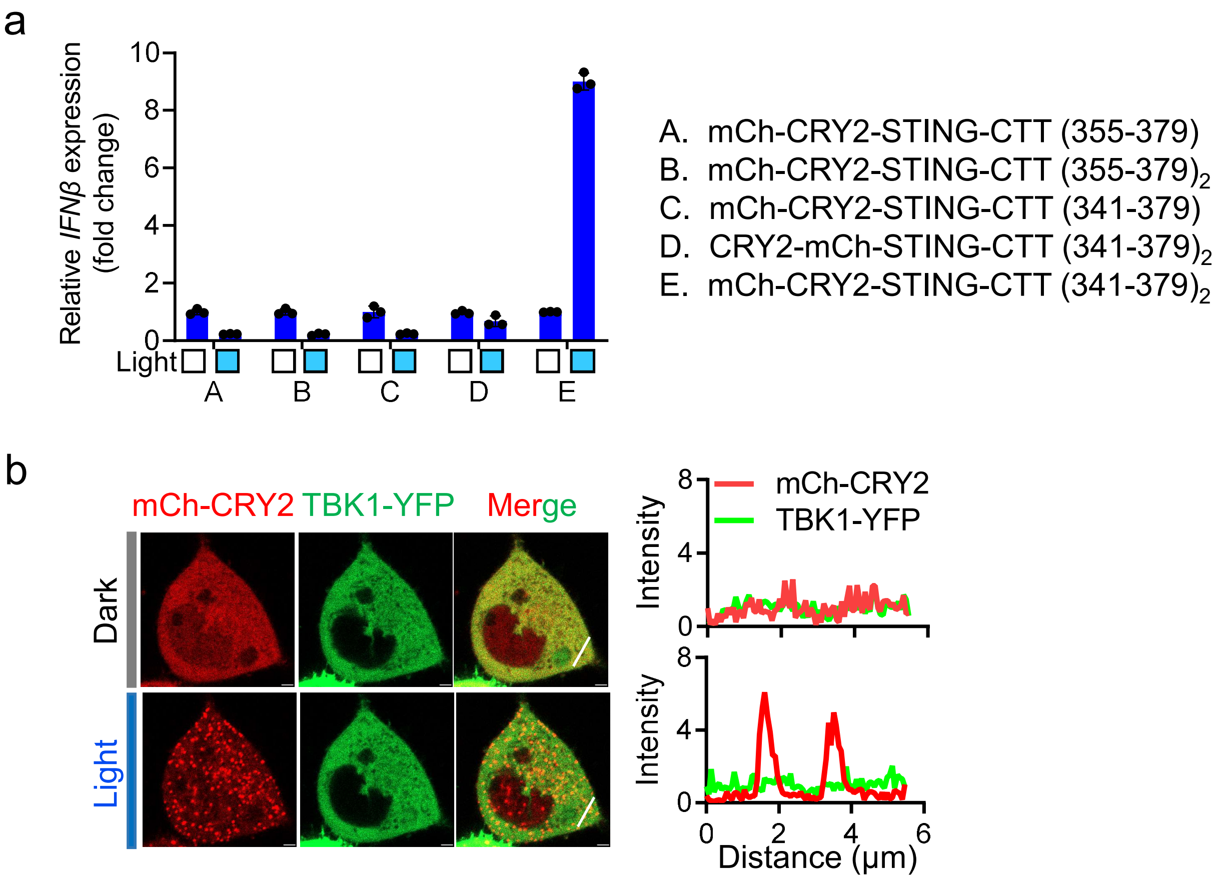

Supplementary Figure 1. Oligomerization of mCh-CRY2 *per se* does not recruit TBK1.

(a) HEK293T cells were transfected with the indicated constructs (A-E), and the expression of *IFNB* was examined after pulsed blue light stimulation (470 nm, 4 mW/cm^2, 30 s ON/OFF cycles for 8 hr; n = 3 independent biological replicates; mean ± S.D.).

(b) Representative confocal images of HeLa cells co-expressing mCh-CRY2 and TBK1-YFP before and after blue light stimulation. The intensity profiles (across the white line) in response to blue light were plotted on the right. Scale bar, 2 μm. Data are from at least three independent experiments. Also see Supplementary Movie 2.

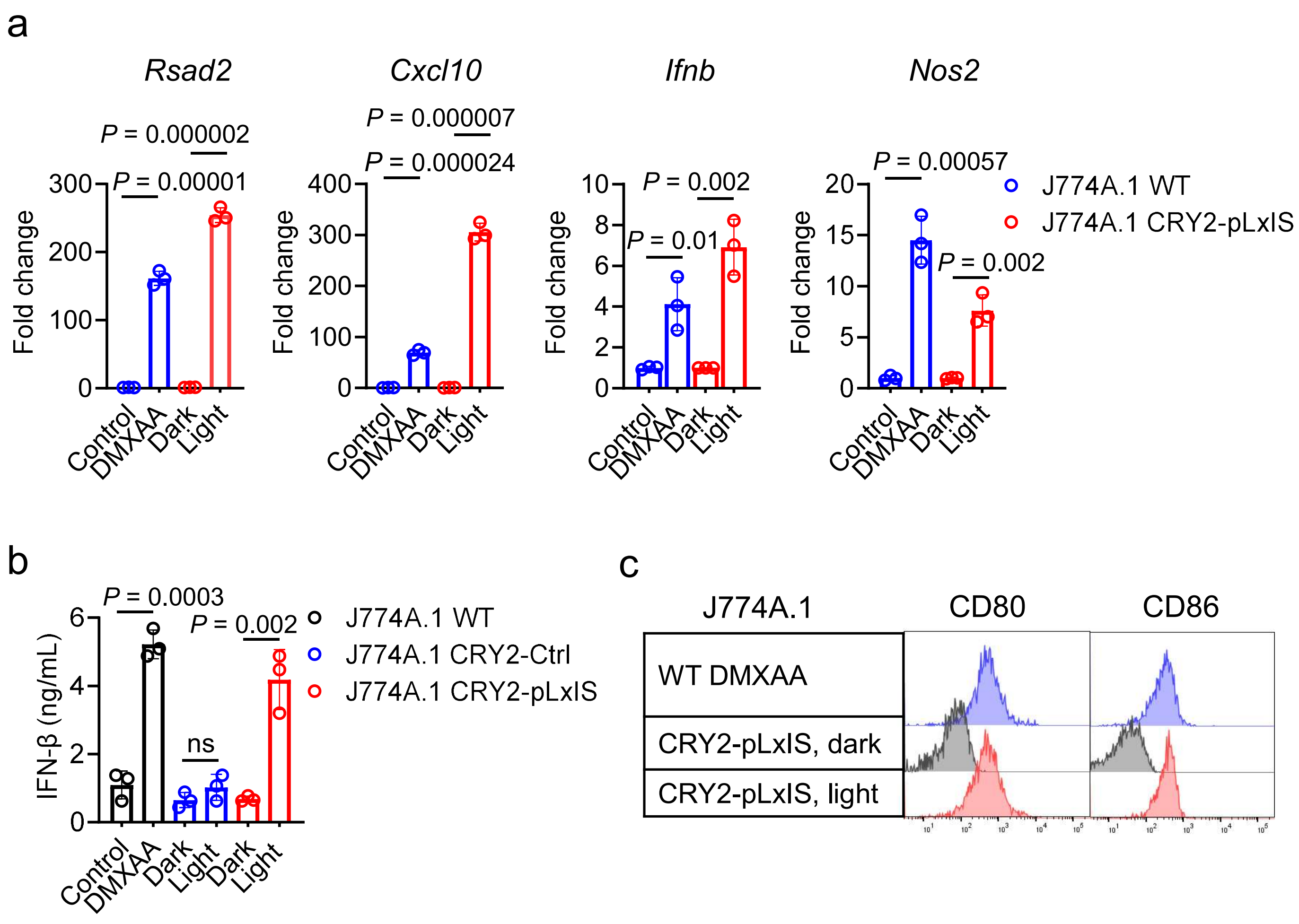

Supplementary Figure 2. Optogenetic control of the STING pathway activation in J774A.1 macrophages.

Wild type (WT) J774A.1 macrophages were treated with a STING agonist DMXAA (20 µg/ml) for 8 hours. J774A.1 macrophages stably expressing CRY2-Ctrl or CRY2-pLxIS were stimulated with pulsed blue light (470 nm, 4 mW/cm^2, 30 s ON/OFF cycles for 8 hr).

(a) Fold change in mRNA expression was determined by qPCR. n = 3 independent biological replicates (mean ± S.D.). Two-sided unpaired Student's t-test.

(b) Quantification of secreted IFN-β under the indicated conditions in (a) by ELISA. n = 3 independent biological replicates (mean ± S.D.; two-sided unpaired Student's t-test).

(c) The expression levels of CD80 and CD86 were examined by flow cytometry 24 hours after DMXAA treatment or blue light stimulation (470 nm, 4 mW/cm^2, 30 s ON/OFF cycles). One representative FACS profile from 3 independent experiments was shown for each condition.

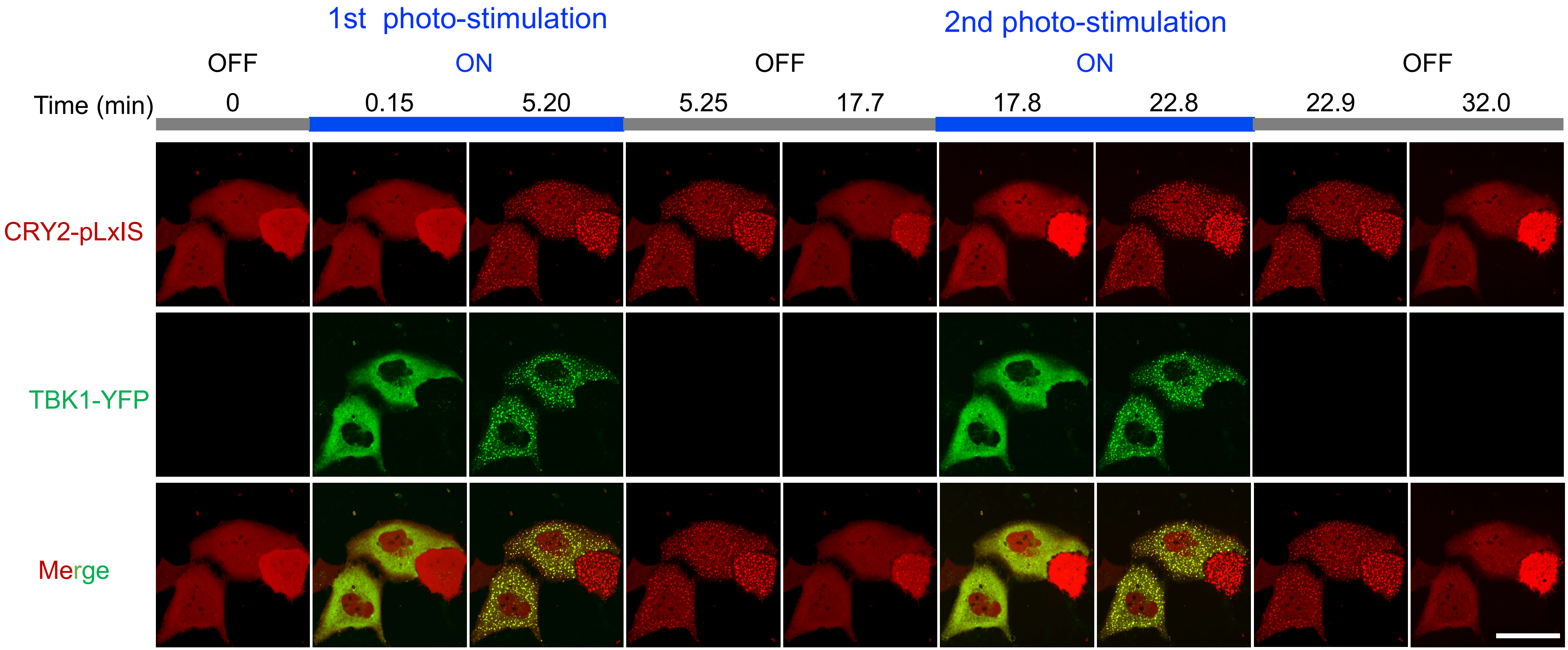

Figure S3. Reversible co-clustering of CRY2-pLxIS with TBK1 in response to two ON/OFF cycles of photo-stimulation. HEK293T cells co-expressing mCherry-CRY2-pLxIS (red) and TBK1-YFP (green) were stimulated by the built-in 488-nm laser source (5% input). Time-lapse confocal imaging showed the reversible clustering of CRY2-pLxIS with subsequent recruitment of TBK1 in response to two ON/OFF cycles of photostimulation (5-sec ON + 10-min OFF). Scale bar, 20 μm. Data represent three independent experiments. Also see Supplementary Movie 3.

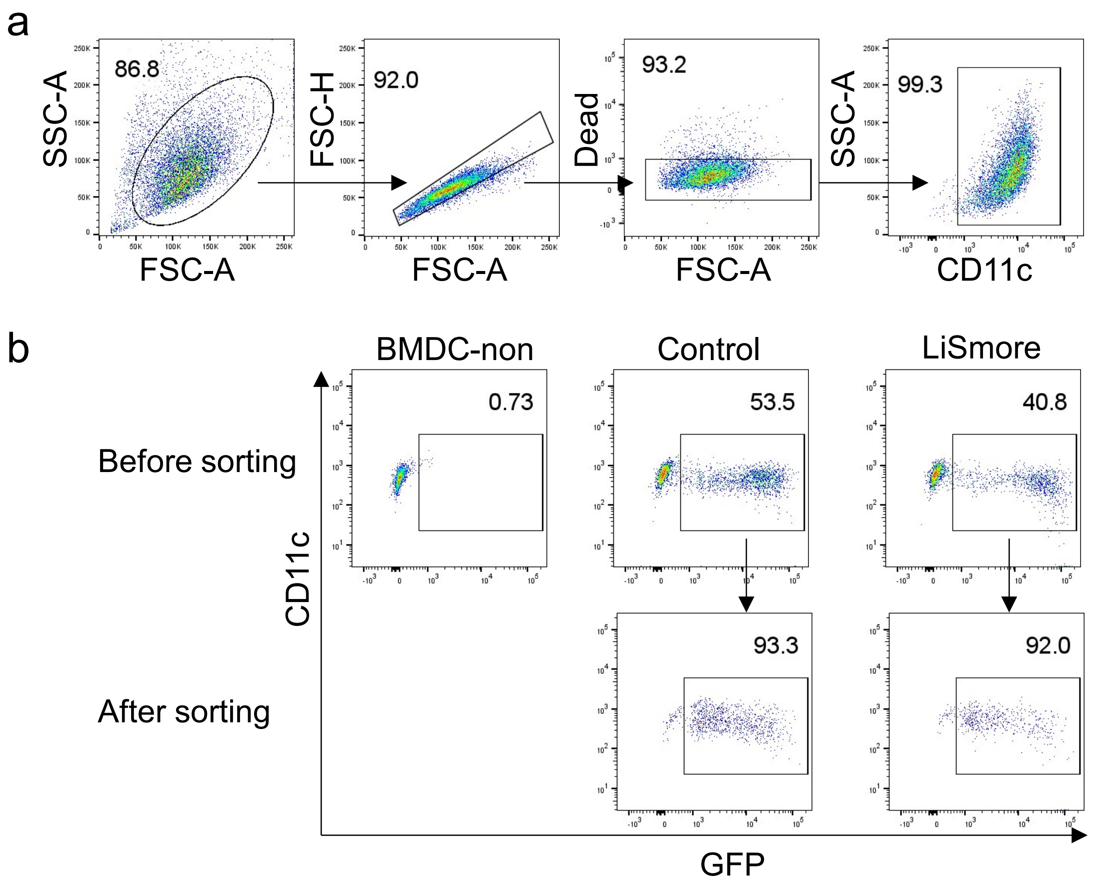

Supplementary Figure 4. The gating strategy for bone-marrow derived dendritic cells (BMDCs).

(a) The gating strategy of flow cytometry analysis to measure live CD11c$^+$ BMDCs. BMDCs were generated in vitro by culturing mouse bone marrow cells in the presence of GM-CSF. BMDCs were then collected on day 6 and seeded into 6-well plates for further transduction of Control (CRY2clust) or LiSmore (CRY2clust-pLxIS).

(b) GFP$^+$CD11c$^+$ BMDCs expressing Control or LiSmore were sorted for both in vitro and in vivo experiments. The percentage of GFP$^+$ population within CD11c$^+$ BMDCs (with a purity of over 90%) before and after sorting was determined by flow cytometry.

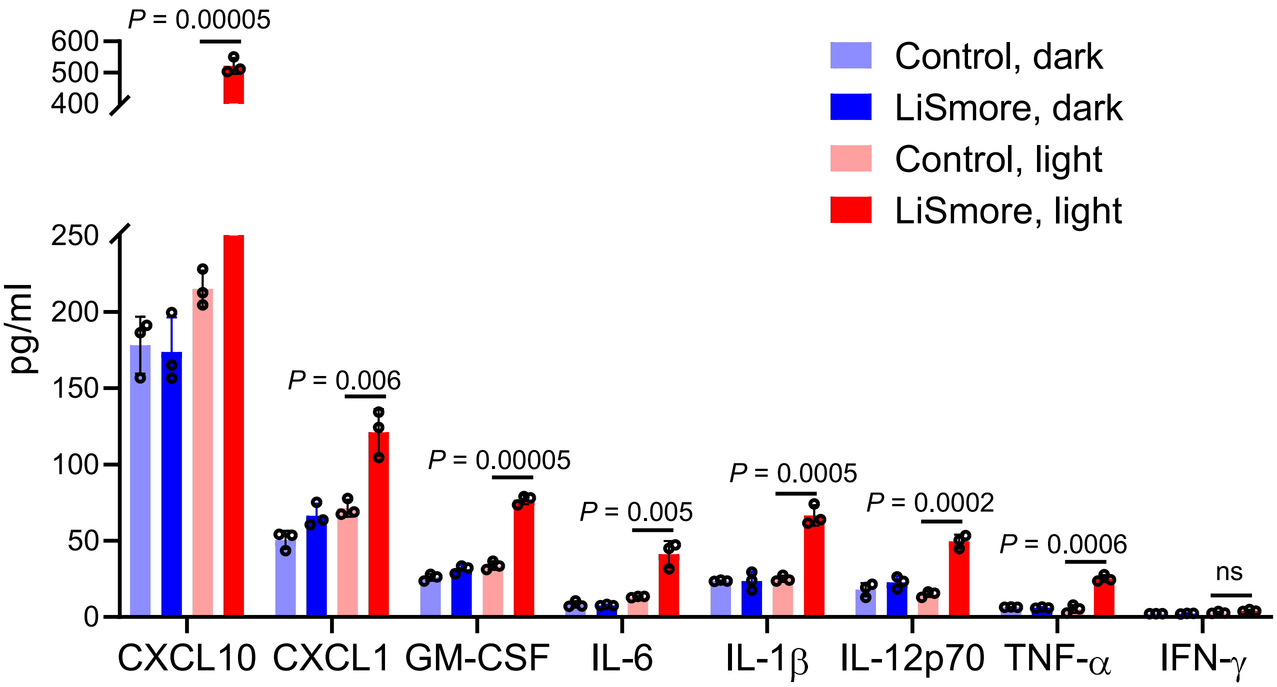

Supplementary Figure 5. LiSmore-BMDCs enable light-inducible production of pro-inflammatory cytokines.

BMDCs were generated in vitro by culturing mouse bone marrow cells in GM-CSF and transduced twice with viruses encoding the Control or LiSmore Constructs. After 36 hours, GFP^+ BMDCs were sorted and then shielded (dark) or exposed to blue light (light; 470 nm, 1 mW/cm^2; 20 s ON, 5 min OFF) in 48-well plates for 18 hours. Concentrations of the indicated cytokines in the supernatants were determined by ELISA (n = 3 independent biological replicates; mean ± S.D.). Two-sided unpaired Student’s t-test.

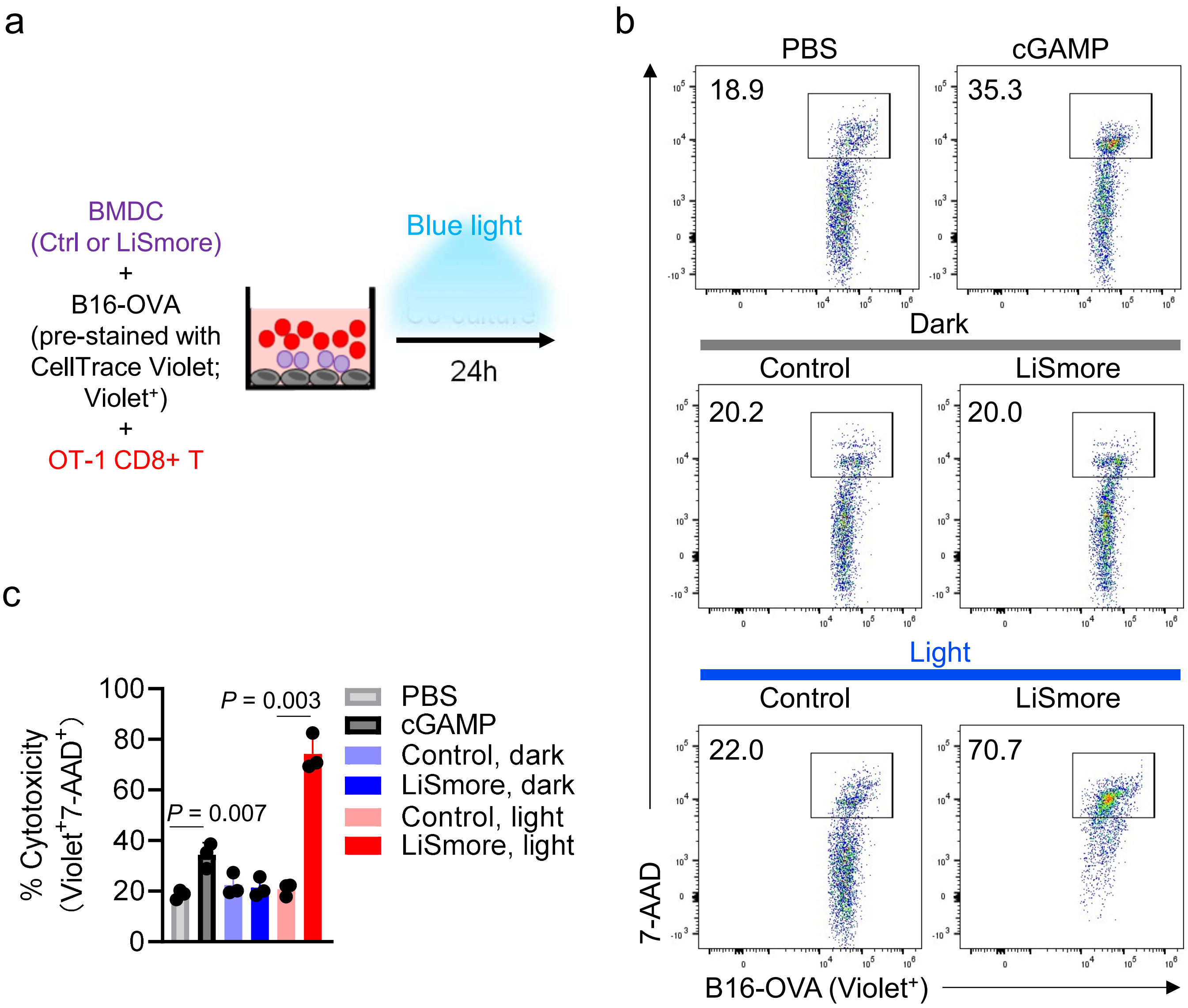

Supplementary Figure 6. Flow cytometry analysis on CD8⁺ T cell-mediated cytotoxicity towards B16-OVA melanoma cells.

(a) Schematic showing the in vitro co-culture system. OT-1 CD8⁺ T cells were incubated with a mixture of LiSmore-BMDCs and B16-OVA cells (pre-stained by CellTrace Violet; Violet⁺) at a 2:1:1 ratio with or without light illumination (470 nm, 1 mW/cm^2; 20 s ON, 5 min OFF; 24 hours). 7-AAD staining was utilized to detect cell death.

(b) Representative FACS profiles for the indicated groups that report B16-OVA melanoma cell death (Violet⁺7-AAD⁺) 24 hours after co-culture.

(c) Quantification of T cell-mediated cytotoxicity toward B16-OVA cells as indicated by the percentage of dead B16-OVA cells (Violet⁺7-AAD⁺). n=3 independent biological replicates (mean ± S.D.). Two-sided unpaired Student's t-test.

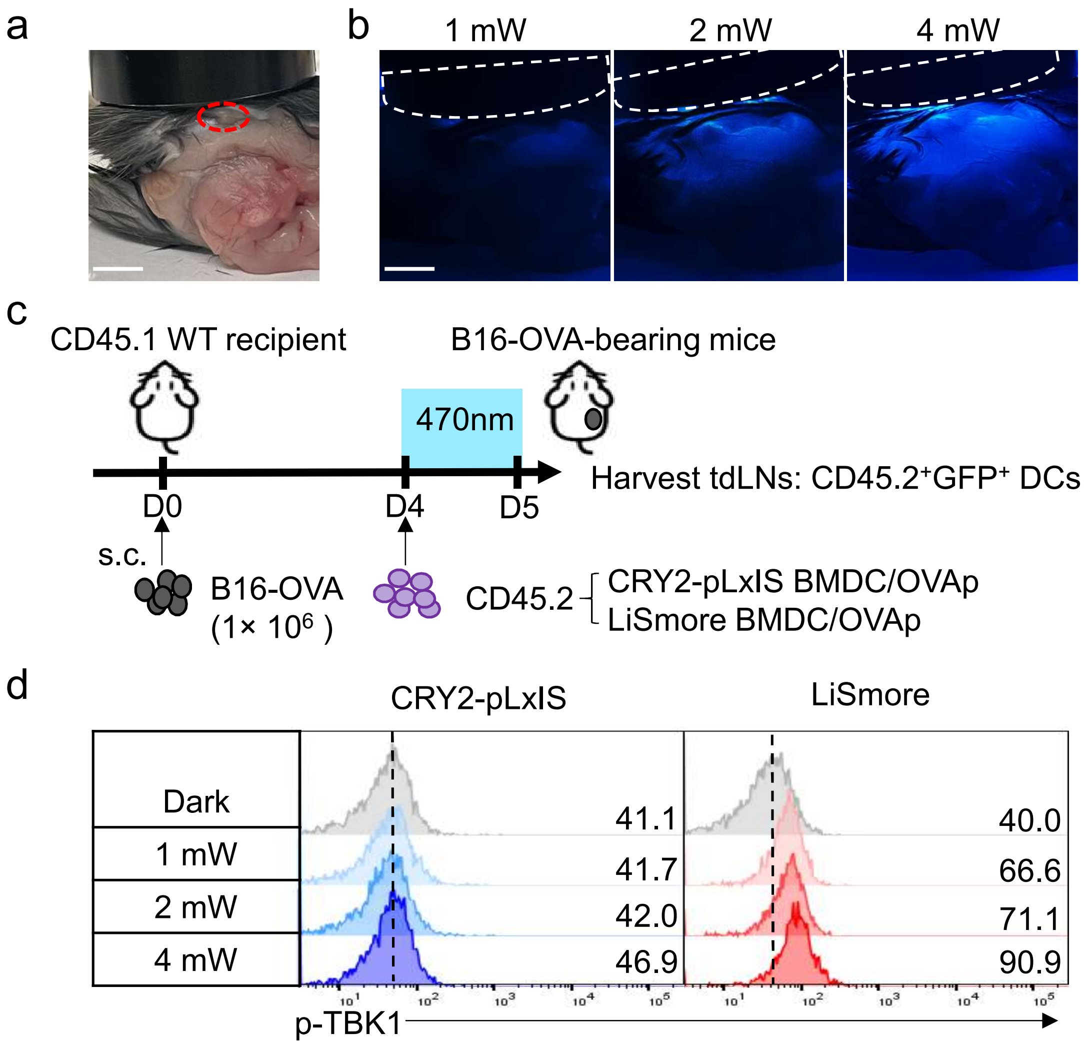

Supplementary Figure 7. Non-invasive optogenetic activation of LiSmore within the mouse inguinal lymph node (ILN) regions from the hind leg.

(a) Side view of blue LED illumination on the dissected mouse flank. Red dotted oval indicated the ILN location under the skin.

(b) Distribution of blue light under the indicated light intensity in the dark field (white dotted line: LED position). Scale bar, 5 mm. Data are representative of 3 independent experiments.

(c) Experimental setup for remote control of STING activation in a B16-OVA melanoma model using non-invasive blue light stimulation. B16-OVA cells (3 x 10^5) were injected (s.c.) in the flank of CD45.1 B6 mice. Mice were transferred with OVA-pulsed CD45.2$^+$ BMDCs that were engineered to express CRY2-pLxIS or LiSmore (CRY2clust-pLxIS) on day 4, and then subjected to pulsed blue light stimulation (light) at the indicated power densities (470 nm; 30 min ON/OFF cycles; 6 hours) or without light stimulation (dark). Transferred dendritic cells (DCs) were isolated from tumor draining lymph nodes (tdLNs) and analyzed by flow cytometry at 18 hours post transfer.

(d) Representative mean fluorescence intensity (MFI) of p-TBK1 staining in CD45.2$^+$GFP$^+$ dendritic cells obtained from the tumor-draining inguinal lymph nodes of mice**.** Data are representative of 3 independent experiments.

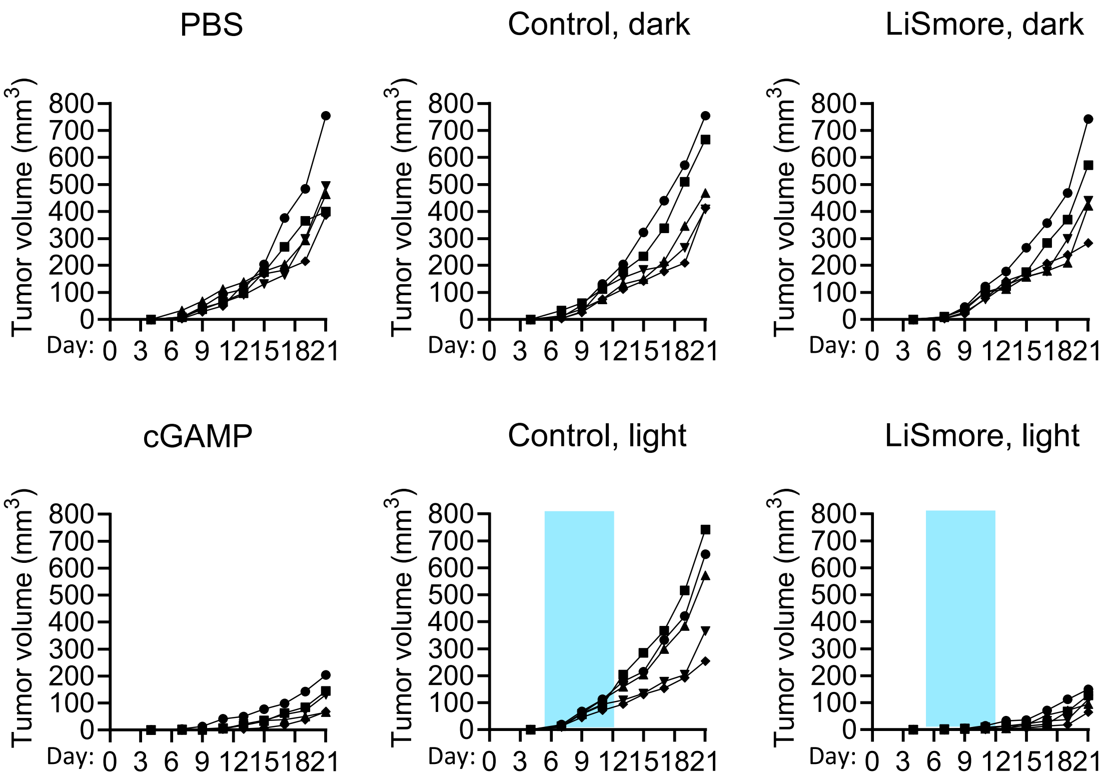

Supplementary Figure 8. Light-induced activation of LiSmore in dendritic cells boosts cytotoxic T lymphocyte (CTL)-mediated antitumor function.

3×10^5 B16-OVA cells were injected (s.c.) in the flank of CD45.1 mice. Mice received PBS, or cGAMP (10 μg/mouse) at days 5, 8 and 11 after tumor inoculation, or were transferred with OVAp-loaded BMDCs expressing Control or LiSmore at day 4, followed by adoptive transfer of $CD45.2^+$ OT-1 $CD8^+$ T cells at day 5. Quantification of individual tumor volume in the indicated experimental groups: PBS, cGAMP treatment, Control without blue light (dark), LiSmore without blue light (dark), Control with blue light (light), and LiSmore with blue light (light), The blue box indicates the time window for photo-stimulation (days 5-12). 470 nm; ~2 mW/cm^2; 30 min ON/OFF cycles for 6 hr per day.

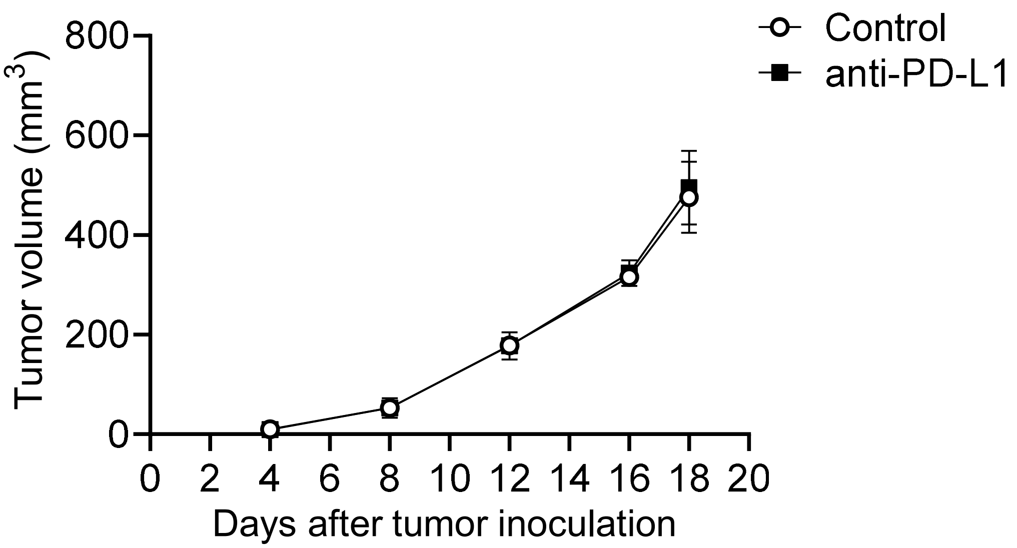

Supplementary Figure 9. The LL/2 tumor does not respond to the anti-PD-L1 treatment. $1×10^6$ LL/2 lung carcinoma cells were injected (s.c.) in the flank of CD45.1 mice. LL/2 tumor cells-implanted mice were intraperitoneally treated with 200 µg of anti-PD-L1 or 200 µg isotype antibody (control) on days 8 and 11. n = 4 biologically independent mice (mean ± S.D.).

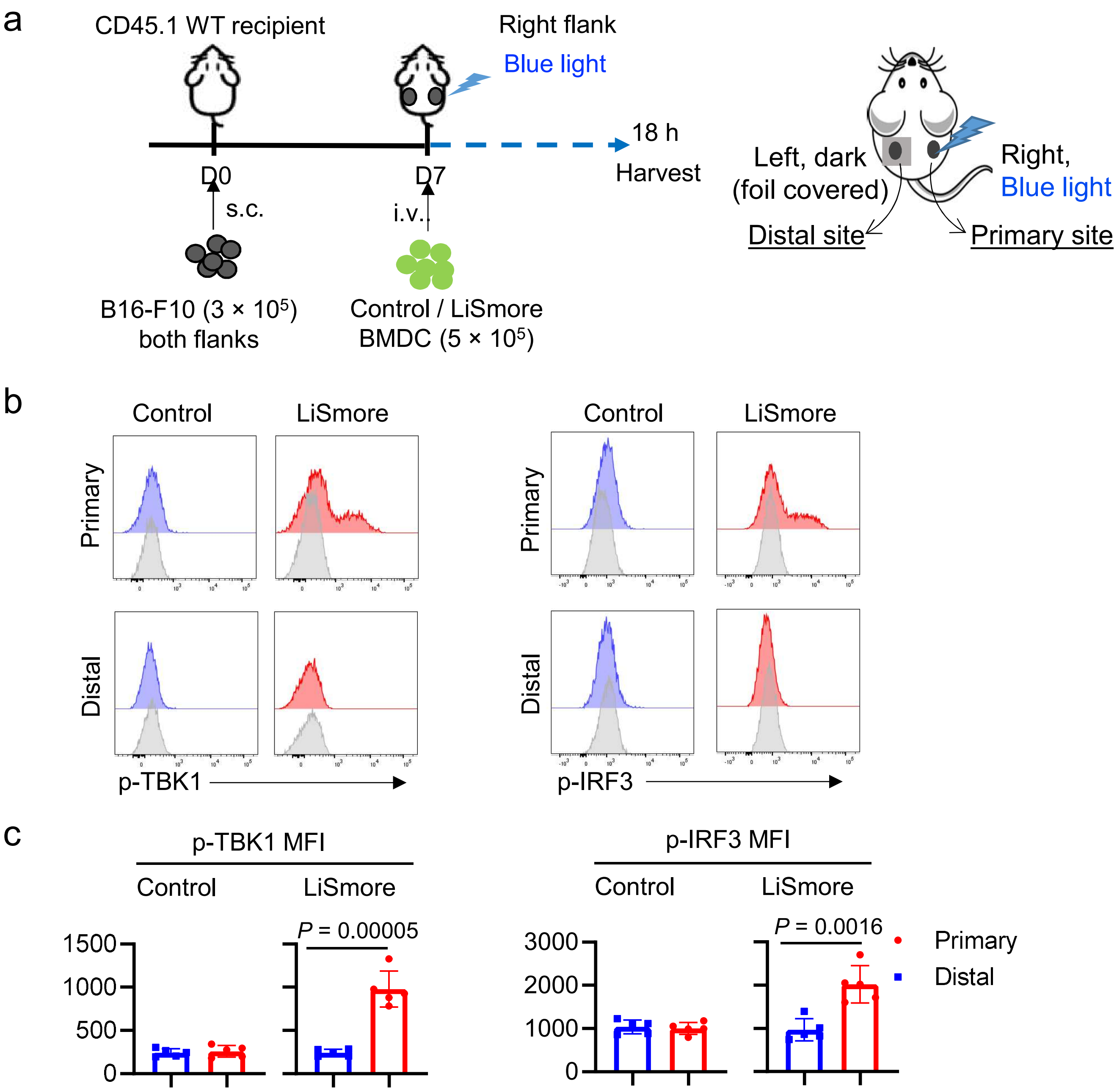

Supplementary Figure 10. Spatial control of LiSmore activation in a bilateral B16-F10 melanoma model.

(a) B16-F10 melanoma cells (3 × 10^5) were inoculated (s.c.) on both flanks of mice for bilateral tumor establishment, followed by injection of engineered BMDCs (Control or LiSmore; 5 × 10^5 cells; i.v.) on day 7. The mice were then subjected to photo-stimulation on the right side of tumor (the primary site; 470 nm; ~2 mW/cm^2; 20 sec ON + 5 min OFF for 18 hr). The left side (the distal site) was shielded from blue light throughout the experiment (dark).

(b) Flow cytometry analysis on the levels of pTBK1 and pIRF3 immunostaining in CD45.2$^+$GFP$^+$ BMDCs isolated from the indicated groups.

(c) Qualification of the mean fluorescence intensity (MFI) of pTBK1 and pIRF3 staining in CD45.2$^+$GFP$^+$ BMDCs. n = 5 biologically independent mice (means ± S.D.; Two-sided unpaired Student's t-test).

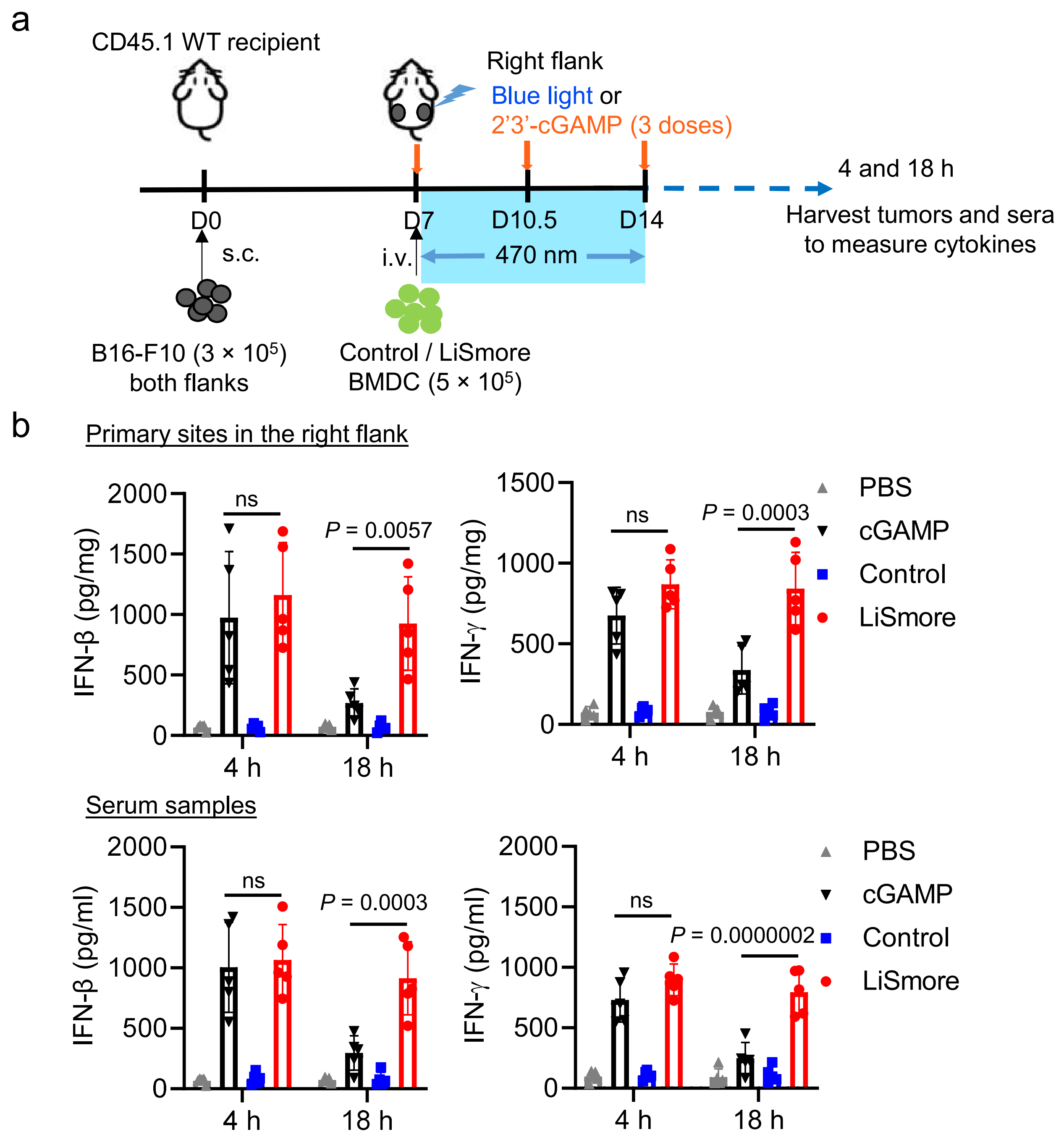

Supplementary Figure 11. Photo-activated LiSmore leads to more sustained production of IFN-β and IFN-γ.

(a) Schematic illustration of the experimental timeline and setup. Both flanks of CD45.1 B6 mice were inoculated with 3 ×10^5 B16-F10 melanoma cells. Mice then received 3 doses intratumoral PBS, or cGAMP (10 μg/mouse) treatment (at days 7, 10.5 and 14) in the right flank, or were transferred with BMDCs expressing Control or LiSmore (5 × 10^5 cells/mouse) at day 7. The right side of the tumor (the primary site) was subjected to photostimulation for 7 days (470 nm; 2 mW/cm^2; 20 sec ON + 5 min OFF; 6 hr per day), while the left side (the distal site) was shielded from blue light (dark).

(b) Quantification of IFN-β and IFN-γ production in the primary tumor sites (top panels) and sera (bottom panels) obtained from bilateral B16-F10 melanoma-bearing mice at 4 and 18 hr after the final treatment. n=5 biologically independent mouse for each group (mean ± S.D.; One-way ANOVA).

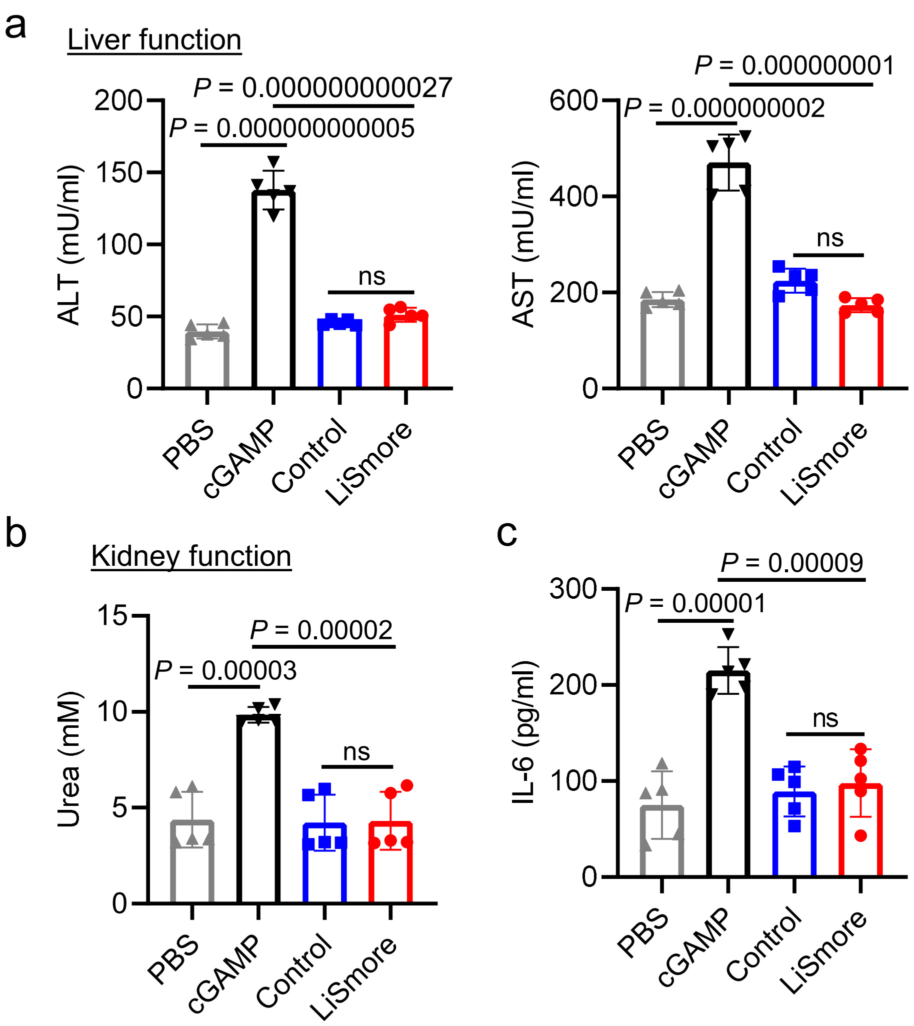

Supplementary Figure 12. Assessment of systemic toxicity after LiSmore-DC or cGAMP treatments. 3 ×10^5 B16-F10 melanoma cells were injected (s.c.) in the right and left flanks of WT CD45.1 B6 mice. Mice received 3 doses intratumoral PBS, or cGAMP (10 µg/mouse) on the right side (day 7, 10.5 and 14), or were transferred with BMDCs expressing Control or LiSmore (5 × 10^5 cells/mouse) at day 7. The right side of the tumor (the primary site) was subjected to pulsed blue light stimulation for 7 days (470 nm; 2 mW/cm^2; 20 sec ON + 5 min OFF; 6 hr per day), while the left side (the distal site) was shielded from blue light (dark). Measurements were conducted one day after the indicated final treatments in the bilateral B16-F10 melanoma-bearing mice (Related to Fig. 6). n=5 biologically independent samples for each group (means ± S.D.; One-way ANOVA).

(a) Quantification of serum concentrations of ALT and AST, which serve as indicators of liver function.

(b) Quantification of serum urea levels, indicative of kidney function.

(c) ELISA measurements of serum levels of IL-6, a key cytokine for assessing systemic inflammation.

Supplementary Table 1. Comparison between LiSmore and cGAMP.

Parameters	cGAMP (STING agonist)	LiSmore (This study)	Notes
**Switches and tissue penetration/ retention**	No switch; No limit for tissue penetration	Blue light-switchable; Ultra-light sensitive; can be activated beneath the skin with 1-4 mW blue light at 470 nm	cGAMP: limited specificity with poor tumor or LNs targeting and retention; LiSmore is compatible with stimulation in deep brain regions or shallow LNs of mice through blue light under low light intensity; Future combination with upconverting nanomaterials can extend the depth of tissue penetration to up to 2-3 cm
**Spatiotemporal control**	Lack of spatial control	Yes	For LiSmore, localized STING pathway activation can be achieved exclusively at photo-stimulated areas
**Reversibility**	No	Yes	LiSmore shows rapid formation of clusters within seconds upon exposure to blue light, and these clusters could be disassembled upon light withdrawal (half-time: ON: ~1-2 min; OFF: ~30 min)
**Abscopal effect**	No	Yes	cGAMP has no appreciable abscopal effect due to its relative short half-life and inability to elicit durable STING activation; LiSmore exhibits noticeable abscopal effect, probably owing to its ability to induce rapid and prolonged STING activation in engineered immune cells
**Systemic toxicity in vivo**	Strong	Weak	cGAMP induces side effects in liver (increase in ALT/AST) and kidney (increase in urea), accompanied with a high serum level of IL-6 (indicative of systemic inflammation); LiSmore-DCs do not seem to cause overt systemic inflammation
**Production and delivery**	Simple; requires multiple dosages via systemic or intratumoral injection	Sophisticated procedures that involve viral transduction and ex vivo culture; administered intratumorally or systemically	Long-lasting STING agonists are needed to minimize the dosage and frequency of injection; The translational potential of LiSmore can be fully unleashed following near-future breakthroughs in in vivo transduction of selected immune cells

Supplementary Table 2. Western blotting antibodies.

Antibody	Company	Clone	Catalog number	Dilution	Validation
Rabbit polyclonal anti-mCherry	Novus Biologicals		NBP2-25157	1:1000	https://www.novusbio.com/products/mcherry-antibody_nbp2-25157
Mouse anti-β-Actin	Santa Cruz		sc-47778	1:1000	https://www.scbt.com/p/beta-actin-antibody-c4?gclid=CjwKCAjwt52mBhB5EiwA05YKox1co62yhvFvNrasIL_wz19PRytCp0McX_xfxaXRvhDB5BRVFl92xBoCQSoQAvD_BwE
Goat anti-mouse IgG–HRP	Santa Cruz		sc-2005	1:1000	https://www.citeab.com/antibodies/3244022-sc-2005-goat-anti-mouse-igg-hrp
Goat anti-rabbit IgG-HRP	Santa Cruz		sc-2004	1:1000	https://www.citeab.com/antibodies/3244042-sc-2004-goat-anti-rabbit-igg-hrp
TBK1/NAK Rabbit mAb	Cell Signaling Technology	D1B4	#3504	1:1000	https://www.cellsignal.com/products/primary-antibodies/tbk1-nak-d1b4-rabbit-mab/3504
Phospho-TBK1/NAK (Ser172) XP Rabbit mAb	Cell Signaling Technology	D52C2	#5483	1:500	https://www.cellsignal.com/products/primary-antibodies/phospho-tbk1-nak-ser172-d52c2-xp-rabbit-mab/5483?site-search-type=Products&N=4294956287&Ntt=%235483&fromPage=plp&_requestid=1340446
IRF-3 XP Rabbit mAb	Cell Signaling Technology	D6I4C	#11904	1:1000	https://www.cellsignal.com/products/primary-antibodies/irf-3-d6i4c-xp-rabbit-mab/11904?site-search-type=Products&N=4294956287&Ntt=%2311904&fromPage=plp&_requestid=1340266
Phospho-IRF-3 (Ser396) Rabbit mAb	Cell Signaling Technology	4D4G	#4947	1:500	https://www.cellsignal.com/products/primary-antibodies/phospho-irf-3-ser396-4d4g-rabbit-mab/4947?site-search-type=Products&N=4294956287&Ntt=%234947&fromPage=plp&_requestid=1340508
NF-κB p65 XP Rabbit mAb	Cell Signaling Technology	D14E12	#8242	1:1000	https://www.cellsignal.com/products/primary-antibodies/nf-kb-p65-d14e12-xp-rabbit-mab/8242?site-search-type=Products&N=4294956287&Ntt=%238242&fromPage=plp&_requestid=1340558
Phospho-NF-κB p65 (Ser536) Rabbit mAb	Cell Signaling Technology	93H1	#3033	1:500	https://www.cellsignal.com/products/primary-antibodies/phospho-nf-kb-p65-ser536-93h1-rabbit-mab/3033?site-search-type=Products&N=4294956287&Ntt=%233033&fromPage=plp&_requestid=1340618

Supplementary Table 3. Flow cytometry antibodies.

Antibody	Company	Clone	Catalog number	Dilution	Validation
anti-CD16/32	BioLegend	93	101302	1:50	https://www.biolegend.com/en-us/products/purified-anti-mouse-cd16-32-antibody-190
PerCP/Cy5.5-CD45	BioLegend	30-F11	103132	1:100	https://www.biolegend.com/en-us/products/percp-cyanine5-5-anti-mouse-cd45-antibody-4264
Alexa Fluor 700-CD45.2	BioLegend	104	109822	1:100	https://www.biolegend.com/en-us/products/alexa-fluor-700-anti-mouse-cd45-2-antibody-3393
Percp-CD11c	BioLegend	N418	117326	1:100	https://www.biolegend.com/en-us/products/percp-anti-mouse-cd11c-antibody-4259
PE-H-2L^{d}/H-2D^{b}	BioLegend	28-14-8	114507	1:100	https://www.biolegend.com/en-us/products/pe-anti-mouse-h-2ld-h-2db-antibody-1690
APC-I-A/I-E	BioLegend	M5/114.15.2	107614	1:100	https://www.biolegend.com/en-us/products/apc-anti-mouse-i-a-i-e-antibody-2488
APC-CD86	BioLegend	GL-1	105012	1:100	https://www.biolegend.com/en-us/products/apc-anti-mouse-cd86-antibody-2896
PE-CD80	BioLegend	16-10A1	104708	1:100	https://www.biolegend.com/en-us/products/pe-anti-mouse-cd80-antibody-43
APC-CD40	BioLegend	3/23	124612	1:100	https://www.biolegend.com/en-us/products/apc-anti-mouse-cd40-antibody-4984
PE-CCR7	BioLegend	4B12	120106	1:100	https://www.biolegend.com/en-us/products/pe-anti-mouse-cd197-ccr7-antibody-2799
PE/Cy7-CD45.1	BioLegend	A20	110730	1:100	https://www.biolegend.com/en-us/products/pe-cyanine7-anti-mouse-cd45-1-antibody-4917
APC-CD8α	BioLegend	53.6-7	100712	1:100	https://www.biolegend.com/en-us/products/apc-anti-mouse-cd8a-antibody-150
PE-CD69	BioLegend	H1.2F3	104508	1:100	https://www.biolegend.com/en-us/products/pe-anti-mouse-cd69-antibody-265

APC anti-human CD80	BioLegend	2D10	305220	1:100	https://www.biolegend.com/en-us/products/apc-anti-human-cd80-antibody-6530
Brilliant Violet 421 anti-human CD86	BioLegend	IT2.2	305426	1:100	https://www.biolegend.com/en-us/products/brilliant-violet-421-anti-human-cd86-antibody-7212
PE-anti-IFN-γ	BioLegend	XMG1.2	505808	1:100	https://www.biolegend.com/en-us/products/pe-anti-mouse-ifn-gamma-antibody-997
PE-anti-Ki67	BioLegend	16A8	652404	1:100	https://www.biolegend.com/en-us/products/pe-anti-mouse-ki-67-antibody-8134
Alexa Fluor 555-p-TBK1	Cell Signaling Technology	D52C2	#70483	1:50	https://www.cellsignal.com/products/antibody-conjugates/phospho-tbk1-nak-ser172-d52c2-xp-rabbit-mab-alexa-fluor-555-conjugate/70483?site-search-type=Products&N=4294956287&Ntt=alexa+fluor%C2%A0555-p-tbk1&fromPage=plp&_requestid=1346147
Alexa Fluor 647-p-IRF3	Cell Signaling Technology	D6O1M	#10327	1:50	https://www.cellsignal.com/products/antibody-conjugates/phospho-irf-3-ser396-d6o1m-rabbit-mab-alexa-fluor-647-conjugate/10327?site-search-type=Products&N=4294956287&Ntt=alexa+fluor%C2%A0647-p-irf3+&fromPage=plp
Alexa Fluor 555-Rabbit mAb IgG	Cell Signaling Technology	DA1E	#3969	1:50	https://www.cellsignal.com/products/antibody-conjugates/rabbit-da1e-mab-igg-xp-isotype-control-alexa-fluor-555-conjugate/3969?site-search-type=Products&N=4294956287&Ntt=+%233969+&fromPage=plp&_requestid=1780643
Alexa Fluor 647-Rabbit mAb IgG	Cell Signaling Technology	DA1E	#2985	1:50	https://www.cellsignal.com/product/productDetail.jsp?productId=2985

Supplementary Table 4. Primer pairs used for real-time PCR analysis.

	Gene	Sequence
Human	*RSAD2 Forward:*	TGGGTGCTTACACCTGCTG
	RSAD2 Reverse:	GAAGTGATAGTTGACGCTGGTT
	CXCL10 Forward:	GTGGCATTCAAGGAGTACCTC
	CXCL10 Reverse:	TGATGGCCTTCGATTCTGGATT
	IFNB Forward:	ATGACCAACAAGTGTCTCCTCC
	IFNB Reverse:	GGAATCCAAGCAAGTTGTAGCTC
	IFIT1 Forward:	TTGATGACGATGAAATGCCTGA
	IFIT1 Reverse:	CAGGTCACCAGACTCCTCAC
	IFIT2 Forward:	AAGCACCTCAAAGGGCAAAAC
	IFIT2 Reverse:	TCGGCCCATGTGATAGTAGAC
	IFIT3 Forward:	TCAGAAGTCTAGTCACTTGGGG
	IFIT3 Reverse:	ACACCTTCGCCCTTTCATTTC
	ISG15 Forward:	CGCAGATCACCCAGAAGATCG
	ISG15 Reverse:	TTCGTCGCATTTGTCCACCA
	ACTB Forward:	TGAAGTGTGACGTGGACATC
	ACTB Reverse:	GGAGGAGCAATGATCTTGAT
Mouse	*Rsad2 Forward:*	GGTGCCTGAATCTAACCAGAAG
	Rsad2 Reverse:	CCACGCCAACATCCAGAATA
	Cxcl10 Forward:	GCCGTCATTTTCTGCCTCA
	Cxcl10 Reverse:	CGTCCTTGCGAGAGGGATC
	Ifnb Forward:	CCCTATGGAGATGACGGAGA
	Ifnb Reverse:	TCCCACGTCAATCTTTCCTC
	Nos2 Forward:	GTTCTCAGCCCAACAATACAAGA
	Nos2 Reverse:	GTGGACGGGTCGATGTCAC
	Hprt Forward:	TCAGTCAACGGGGGACATAAA
	Hprt Reverse:	GGGGCTGTACTGCTTAACCAG
