# Supplementary material for: Optogenetic engineering of STING signaling allows remote immunomodulation to enhance cancer immunotherapy
Source: Nat Commun. 2023 Sep 6;14:5461. doi: 10.1038/s41467-023-41164-2 (PMC10482946; doi:10.1038/s41467-023-41164-2)
Supplement: Supplementary file 3 — Reporting Summary [file 41467_2023_41164_MOESM3_ESM.pdf]

nature portfolio

Corresponding author(s): Yubin Zhou

Last updated by author(s): Aug 7, 2023

Reporting Summary

Nature Portfolio wishes to improve the reproducibility of the work that we publish. This form provides structure for consistency and transparency in reporting. For further information on Nature Portfolio policies, see our Editorial Policies and the Editorial Policy Checklist.

Statistics

For all statistical analyses, confirm that the following items are present in the figure legend, table legend, main text, or Methods section.

n/a	Confirmed	
☐	☒	The exact sample size (*n*) for each experimental group/condition, given as a discrete number and unit of measurement
☐	☒	A statement on whether measurements were taken from distinct samples or whether the same sample was measured repeatedly
☐	☒	The statistical test(s) used AND whether they are one- or two-sided<br>*Only common tests should be described solely by name; describe more complex techniques in the Methods section.*
☒	☐	A description of all covariates tested
☒	☐	A description of any assumptions or corrections, such as tests of normality and adjustment for multiple comparisons
☐	☒	A full description of the statistical parameters including central tendency (e.g. means) or other basic estimates (e.g. regression coefficient) AND variation (e.g. standard deviation) or associated estimates of uncertainty (e.g. confidence intervals)
☐	☒	For null hypothesis testing, the test statistic (e.g. *F*, *t*, *r*) with confidence intervals, effect sizes, degrees of freedom and *P* value noted<br>*Give P values as exact values whenever suitable.*
☒	☐	For Bayesian analysis, information on the choice of priors and Markov chain Monte Carlo settings
☒	☐	For hierarchical and complex designs, identification of the appropriate level for tests and full reporting of outcomes
☒	☐	Estimates of effect sizes (e.g. Cohen's *d*, Pearson's *r*), indicating how they were calculated

Our web collection on statistics for biologists contains articles on many of the points above.

Software and code

Policy information about availability of computer code

Data collection	The live cell imaging data were obtained by using a Nikon NIS-Elements AR cofocal imaging system (Nikon, version 4.51.00). The FACS data were obtained using an LSRII cytometer (BD Biosciences, Franklin Lakes, NJ, USA), with cell sorting conducted by a FACSFusion cell sorter (BD Biosciences. Franklin Lakes, NJ, USA). The ELISA absorbance was recorded by using a Synergy Neo2 plate reader (BioTek, Winooski, VT, USA). The antigen-antibody complexes during immunoblotting were visualized through the ChemiDoc Imaging System (Bio-Rad, Hercules, CA, USA). Real-time PCR was performed using an ABI PRISM cycler (Life Technologies, Carlsbad, CA, USA). Concept images was created by Biorender.com.
Data analysis	Imaging data analyses were performed using the Nikon NIS-Elements AR Analysis (version 4.51.00) or the ImageJ program (NIH, version 1.53k). FlowJo (v10.5.3) was used for all flow cytometry analysis. GraphPad Prism7 was used to generate all graphs and to perform statistical analyses. Statistical analysis was performed using either One-Way ANOVA or two-tailed unpaired Student's t-test whenever appropriate.

For manuscripts utilizing custom algorithms or software that are central to the research but not yet described in published literature, software must be made available to editors and reviewers. We strongly encourage code deposition in a community repository (e.g. GitHub). See the Nature Portfolio guidelines for submitting code & software for further information.

Data

Policy information about availability of data

All manuscripts must include a data availability statement. This statement should provide the following information, where applicable:

- Accession codes, unique identifiers, or web links for publicly available datasets
- A description of any restrictions on data availability
- For clinical datasets or third party data, please ensure that the statement adheres to our policy

The authors declare that the data supporting the findings of this study are available within the paper and its supplementary information files. The plasmids and all other data are available from the corresponding author upon reasonable request. Source data are provided with this paper.

Human research participants

Policy information about studies involving human research participants and Sex and Gender in Research.

Reporting on sex and gender	No human research involved.
Population characteristics	No human research involved.
Recruitment	No human research involved.
Ethics oversight	No human research involved.

Note that full information on the approval of the study protocol must also be provided in the manuscript.

Field-specific reporting

Please select the one below that is the best fit for your research. If you are not sure, read the appropriate sections before making your selection.

☒ Life sciences ☐ Behavioural & social sciences ☐ Ecological, evolutionary & environmental sciences

For a reference copy of the document with all sections, see nature.com/documents/nr-reporting-summary-flat.pdf

Life sciences study design

All studies must disclose on these points even when the disclosure is negative.

Sample size	No statistical tests were performed to predetermine the sample sizes. Sample sizes were chosen on the basis of an initial pilot experiment and further based on similar experiments reported in previous publications, "Ma, G., He, L., Liu, S. et al. Optogenetic engineering to probe the molecular choreography of STIM1-mediated cell signaling. Nat Commun 11, 1039 (2020)". At least 3 biological replicates were performed for most cases, unless otherwise noted in the figure legends.
Data exclusions	No data were excluded from the analysis.
Replication	All the experiments were successfully replicated three times. The number of replicates performed is indicated in each figure legend, where applicable.
Randomization	All the samples and controls were treated side-by-side using the identical protocols and the samples in the same experiment group were random. The detected cells or imaging views were randomly selected.
Blinding	The investigators were blinded to group allocation during data collection and analysis.

Reporting for specific materials, systems and methods

We require information from authors about some types of materials, experimental systems and methods used in many studies. Here, indicate whether each material, system or method listed is relevant to your study. If you are not sure if a list item applies to your research, read the appropriate section before selecting a response.

Materials & experimental systems

n/a	Involved in the study
☐	☒ Antibodies
☐	☒ Eukaryotic cell lines
☒	☐ Palaeontology and archaeology
☐	☒ Animals and other organisms
☒	☐ Clinical data
☒	☐ Dual use research of concern

Methods

n/a	Involved in the study
☒	☐ ChIP-seq
☐	☒ Flow cytometry
☒	☐ MRI-based neuroimaging

Antibodies

Antibodies used

The following primary antibodies were used for immunoblotting:
The rabbit polyclonal anti-mCherry, NBP2-25157, Novus Biologicals (Littleton, Colorado, USA), 1:1000.
The mouse anti-β-Actin, sc-47778, Santa Cruz Biotechnology (Dallas, TX, USA), 1:1000.
The goat anti-mouse IgG–HRP, sc-2005, Santa Cruz Biotechnology (Dallas, TX, USA), 1:1000.
The goat anti-rabbit IgG-HRP, sc-2004, Santa Cruz Biotechnology (Dallas, TX, USA), 1:1000.
TBK1/NAK (D1B4) Rabbit mAb, #3504, Cell Signaling Technology (Danvers, MA, USA), 1:1000.
Phospho-TBK1/NAK (Ser172) (D52C2) XP Rabbit mAb, #5483, Cell Signaling Technology (Danvers, MA, USA), 1:500.
IRF-3 (D6I4C) XP Rabbit mAb, #11904, Cell Signaling Technology (Danvers, MA, USA), 1:1000.
Phospho-IRF-3 (Ser396) (4D4G) Rabbit mAb, #4947, Cell Signaling Technology (Danvers, MA, USA), 1:500.
NF-κB p65 (D14E12) XP Rabbit mAb, #8242, Cell Signaling Technology (Danvers, MA, USA), 1:1000.
Phospho-NF-κB p65 (Ser536) (93H1) Rabbit mAb, #3033, Cell Signaling Technology (Danvers, MA, USA), 1:500.

The following Abs were used for FACS:
Phospho-TBK1/NAK (Ser172) (D52C2) XP Rabbit mAb (Alexa Fluor 555), #70483, Cell Signaling Technology (Danvers, MA, USA), 1:50.
Phospho-IRF-3 (Ser396) (D6O1M) Rabbit mAb (Alexa Fluor 647), #10327, Cell Signaling Technology (Danvers, MA, USA), 1:50.
Rabbit (DA1E) mAb IgG XP Isotype Control (Alexa Fluor 555) #3969, Cell Signaling Technology (Danvers, MA, USA), 1:50.
Rabbit (DA1E) mAb IgG XP Isotype Control (Alexa Fluor 647), #2985, Cell Signaling Technology (Danvers, MA, USA), 1:50.

The following Abs used for FACS (all from BioLegend Inc):
Purified anti-mouse CD16/32 Antibody (93), #101302, 1:50.
PerCP/Cy5.5-CD45 (30-F11), #103132, 1:100.
Alexa Fluor 700-CD45.2 (104), #109822, 1:100.
Percp-CD11c (N418), #117326, 1:100.
PE-H2Kb (28-14-8), #114507, 1:100.
APC-IA/IE (M5/114.15.2), #107614, 1:100.
APC-CD86 (GL-1), #105012, 1:100.
PE-CD80 (16-10A1), #104708, 1:100.
APC-CD40 (3/23), #124612, 1:100.
PE-CCR7 (4B12), #120106, 1:100.
PE/Cy7-CD45.1 (A20), #110730, 1:100.
APC-CD8α (53.6-7), #100712, 1:100.
PE-CD69 (H1.2F3), #104508, 1:100.
APC anti-human CD80 (2D10), #305220, 1:100.
Brilliant Violet 421 anti-human CD86 (IT2.2), #305426, 1:100.
PE-anti-Ki67 (16A8), #652404, 1:100.
PE-anti-IFN-γ (XMG1.2), #505808, 1:100.

Validation

All antibodies were verified by the supplier and each lot has been quality tested.
Antibody Validations are defined as follows:
Rabbit polyclonal anti-mCherry, NBP2-25157, https://www.novusbio.com/products/mcherry-antibody_nbp2-25157.
Mouse anti-β-Actin, sc-47778, https://www.scbt.com/p/beta-actin-antibody-c4?gclid=CjwKCAjwt52mBhB5EiwA05YKox1co62yhvFvNrasIL_wz19PRytCp0McX_xfxaXRvhDB5BRVFl92xBoCQSoQAvD_BwE.
Goat anti-mouse IgG–HRP, sc-2005, https://www.citeab.com/antibodies/3244022-sc-2005-goat-anti-mouse-igg-hrp.
Goat anti-rabbit IgG-HRP, sc-2004, https://www.citeab.com/antibodies/3244042-sc-2004-goat-anti-rabbit-igg-hrp.
TBK1/NAK Rabbit, #3504, https://www.cellsignal.com/products/primary-antibodies/tbk1-nak-d1b4-rabbit-mab/3504.
Phospho-TBK1/NAK (Ser172) XP Rabbit mAb, #5483, https://www.cellsignal.com/products/primary-antibodies/phospho-tbk1-nak-ser172-d52c2-xp-rabbit-mab/5483?site-search-type=Products&N=4294956287&Ntt=%235483&fromPage=plp&_requestid=1340446.
IRF-3 XP Rabbit mAb, #11904, https://www.cellsignal.com/products/primary-antibodies/irf-3-d6i4c-xp-rabbit-mab/11904?site-search-type=Products&N=4294956287&Ntt=%2311904&fromPage=plp&_requestid=1340266.
Phospho-IRF-3 (Ser396) Rabbit mAb, #4947, https://www.cellsignal.com/products/primary-antibodies/phospho-irf-3-ser396-4d4g-rabbit-mab/4947?site-search-type=Products&N=4294956287&Ntt=%234947&fromPage=plp&_requestid=1340508.
NF-κB p65 XP Rabbit mAb, #8242, https://www.cellsignal.com/products/primary-antibodies/nf-kb-p65-d14e12-xp-rabbit-mab/8242?site-search-type=Products&N=4294956287&Ntt=%238242&fromPage=plp&_requestid=1340558.
Phospho-NF-κB p65 (Ser536) Rabbit mAb, #3033, https://www.cellsignal.com/products/primary-antibodies/phospho-nf-kb-p65-ser536-93h1-rabbit-mab/3033?site-search-type=Products&N=4294956287&Ntt=%233033&fromPage=plp&_requestid=1340618.

Anti-CD16/32, #101302, https://www.biolegend.com/en-us/products/purified-anti-mouse-cd16-32-antibody-190.
PerCP/Cy5.5-CD45, #103132, https://www.biolegend.com/en-us/products/percp-cyanine5-5-anti-mouse-cd45-antibody-4264
Alexa Fluor 700-CD45.2 BioLegend 104 109822 1:100 https://www.biolegend.com/en-us/products/alexa-fluor-700-anti-mouse-

cd45-2-antibody-3393.
Percp-CD11c, #117326, https://www.biolegend.com/en-us/products/percp-anti-mouse-cd11c-antibody-4259.
PE-H-2Ld/H-2Db, #114507, https://www.biolegend.com/en-us/products/pe-anti-mouse-h-2ld-h-2db-antibody-1690.
APC-I-A/I-E, #107614, https://www.biolegend.com/en-us/products/apc-anti-mouse-i-a-i-e-antibody-2488.
APC-CD86, #105012, https://www.biolegend.com/en-us/products/apc-anti-mouse-cd86-antibody-2896.
PE-CD80, #104708, https://www.biolegend.com/en-us/products/pe-anti-mouse-cd80-antibody-43.
APC-CD40, #124612, https://www.biolegend.com/en-us/products/apc-anti-mouse-cd40-antibody-4984.
PE-CCR7, #120106, https://www.biolegend.com/en-us/products/pe-anti-mouse-cd197-ccr7-antibody-2799.
PE/Cy7-CD45.1, #110730, https://www.biolegend.com/en-us/products/pe-cyanine7-anti-mouse-cd45-1-antibody-4917.
APC-CD8α, #100712, https://www.biolegend.com/en-us/products/apc-anti-mouse-cd8a-antibody-150.
PE-CD69, #104508, https://www.biolegend.com/en-us/products/pe-anti-mouse-cd69-antibody-265.
APC anti-human CD80, #305220, https://www.biolegend.com/en-us/products/apc-anti-human-cd80-antibody-6530.
Brilliant Violet 421 anti-human CD86, #305426, https://www.biolegend.com/en-us/products/brilliant-violet-421-anti-human-cd86-antibody-7212.
PE-anti-IFN-γ, #505808, https://www.biolegend.com/en-us/products/pe-anti-mouse-ifn-gamma-antibody-997.
PE-anti-Ki67, #652404, https://www.biolegend.com/en-us/products/pe-anti-mouse-ki-67-antibody-8134.
Alexa Fluor 555-p-TBK1, #70483, https://www.cellsignal.com/products/antibody-conjugates/phospho-tbk1-nak-ser172-d52c2-xp-rabbit-mab-alexa-fluor-555-conjugate/70483?site-search-type=Products&N=4294956287&Ntt=alexa+fluor%C2%A0555-p-tbk1&fromPage=plp&_requestid=1346147.
Alexa Fluor 647-p-IRF3, #10327, https://www.cellsignal.com/products/antibody-conjugates/phospho-irf-3-ser396-d6o1m-rabbit-mab-alexa-fluor-647-conjugate/10327?site-search-type=Products&N=4294956287&Ntt=alexa+fluor%C2%A0647-p-irf3+&fromPage=plp.
Alexa Fluor 555-Rabbit mAb IgG, #3969, https://www.cellsignal.com/products/antibody-conjugates/rabbit-da1e-mab-igg-xp-isotype-control-alexa-fluor-555-conjugate/3969?site-search-type=Products&N=4294956287&Ntt=+%233969+&fromPage=plp&_requestid=1780643.
Alexa Fluor 647-Rabbit mAb IgG, #2985, https://www.cellsignal.com/product/productDetail.jsp?productId=2985.

Eukaryotic cell lines

Policy information about cell lines and Sex and Gender in Research

Cell line source(s)	HeLa, HEK293T, THP-1, J774A.1 were obtained from the American Type Culture Collection (ATCC, Manassas, VA, USA). B16F10/OVA was a gift from Dr. Anjana Rao at La Jolla Institute for Immunology. LL/2 cells stably expressing OVA was generated by our lab. Plat-E cells were from Cell Biolabs (#RV-101; San Diego, California, USA).
Authentication	Authenticated by the vendors with STR profiling.
Mycoplasma contamination	Mycoplasma contamination was tested by using the LookOut PCR detection kit from SigmaAldrich. All cell lines used were mycoplasma free.
Commonly misidentified lines (See ICLAC register)	No commonly misidentified cell lines were used.

Animals and other research organisms

Policy information about studies involving animals; ARRIVE guidelines recommended for reporting animal research, and Sex and Gender in Research

Laboratory animals	6~8 weeks of C57BL/6-Tg (TcraTcrb) 1100Mjb/J (CD45.2, H-2b) (OT-I) mice (Strain #: 003831), C57BL/6-CD45.1 (B6 CD45.1, Strain #: 002014) and C57BL/6J (B6 CD45.2, Strain #: 000664) mice were purchased from the Jackson laboratory (Bar Harbor, ME, USA). All mice were maintained in the animal facility at the Institute of Biosciences and Technology, Texas A&M University (Houston, TX, USA) in specific pathogen-free/SPF conditions under standard conditions (23~26°C, 40%~60% humidity, 12 h light-dark cycle).
Wild animals	The study did not involve wild animals.
Reporting on sex	Both males and females were used in the study.
Field-collected samples	The study did not involve samples collected from the field.
Ethics oversight	All animal studies were approved by the Institutional Animal Care Use Committee (IACUC) of the Texas A&M University Institute of Biosciences and Technology with the maximum allowable tumor size of <20 mm in diameter for each mouse (Protocol # IACUC 2021-01850-IBT).

Note that full information on the approval of the study protocol must also be provided in the manuscript.

Flow Cytometry

Plots

Confirm that:

☒ The axis labels state the marker and fluorochrome used (e.g. CD4-FITC).

☒ The axis scales are clearly visible. Include numbers along axes only for bottom left plot of group (a 'group' is an analysis of identical markers).

☒ All plots are contour plots with outliers or pseudocolor plots.

☒ A numerical value for number of cells or percentage (with statistics) is provided.

Methodology

Sample preparation	All cells were cultured according to the standard protocols from the distributors. Doublets and debris of dead cells were excluded before gating.
Instrument	All data were collected using the LSRII flow cytometer (BD Biosciences, Franklin Lakes, NJ, USA).
Software	BD FlowJo (v10.5.3)
Cell population abundance	CD11c+ abundance is ~90% of the live BMDC population.<br>GFP+CD11c+ DCs were sorted to >90% purity.
Gating strategy	Cells were gated by FSC/SSC gates and the FSC/FSC-width to select single cells. Cells were stained with Zombie Aqua Fixable viability Kit (Biolegend, San Diego, CA, USA, #423102) to discriminate live and dead cells. After that positive cell boundaries was performed by comparision with matched isotyoe controls and unstained controls. The gating strategy was provided in the Supplementary figures.

☒ Tick this box to confirm that a figure exemplifying the gating strategy is provided in the Supplementary Information.
