# Supplementary material for: Optogenetic engineering of STING signaling allows remote immunomodulation to enhance cancer immunotherapy
Source: Nat Commun. 2023 Sep 6;14:5461. doi: 10.1038/s41467-023-41164-2 (PMC10482946; doi:10.1038/s41467-023-41164-2)
Supplement: Supplementary file 4 — Description of additional supplementary files [file 41467_2023_41164_MOESM4_ESM.pdf]

Captions for Supplementary Movies

Supplementary Movie 1. Light-induced co-clustering of TBK1 (green) with CRY2-pLxIS (red) in HeLa cells.

HeLa cells transfected with plasmids encoding mCh-CRY2-pLxIS (red; left) and TBK1-YFP (green; middle) were subjected to photostimulation for 5 min at 5-second intervals (488 nm, 5% output). Scale bar, 10 μm. Also see Fig. 1c.

Supplementary Movie 2. Light-induced oligomerization of mCh-CRY2 (red) did not show colocalization with TBK1 (green) in HeLa cells.

HeLa cells transfected with plasmids encoding mCh-CRY2 (red; left) and TBK1-YFP (green; middle) were photo-stimulated for 5 min at 5-second intervals (488 nm, 5% output). Scale bar, 10 μm. Also see Supplementary Fig. 1b.

Supplementary Movie 3. Reversible co-clustering of CRY2-pLxIS (red) with TBK1 (green) in response to repeated ON/OFF cycles of photostimulation.

HeLa cells co-expressing mCh-CRY2-pLxIS (red; left) and TBK1-YFP (green; middle) were subjected to two ON/OFF cycles of light stimulation (5-sec ON +10-min OFF; 488 nm, 5% output). Note: the GFP channel was turned off during the imaging to allow CRY2-pLxIS returning to its dark state, hence no fluorescence signal for TBK1-YFP during the dark period. Also see Supplementary Fig. 3.
